# Cryptococcal cerebellitis in no-VIH patient

**Published:** 2017-06-30

**Authors:** Fabricio Andres Lasso, Tomas Omar Zamora Bastidas, Jorge Andrés Potosí García, Bairon Díaz Idrobo

**Affiliations:** 1 Facultad de Medicina, Universidad del Cauca. Cauca, Colombia; 2 Medicina Interna. Facultad de Medicina. Universidad del Cauca. Cauca, Colombia

**Keywords:** Cerebellar diseases, cryptococcal, meningitis, Cryptococcus, adult, age, Immunocompetence, Cryptococcosis, Cryptococcus neofromans

## Abstract

**Introduction:**

Cryptococcosis is an opportunistic fungal infection whose etiology is *Cryptococcus neofromans / C. gattii,* complex which affects immunocompromised patients mainly. Meningeal infection is one of the most common presentations, but cerebellar affection is rare.

**Case Description:**

Male patient with 65 old years, from an area of subtropical climate with chronic exposure to poultry, without pathological antecedents, who presented clinical picture consistent with headache, fever, seizures and altered mental status.

**Clinical findings and diagnostic methods:**

Initially without menigeal signs or intracranial hypertension and normal neurological examination. Later, the patient developed ataxia, dysdiadochokinesia and limb loss. By lumbar punction and image of nuclear magnetic resonance (NMR) cerebellitis cryptococcal was diagnosticated.

**Treatment:**

Antifungal therapy with amphotericin B and fluconazole was performed, however the patient died.

**Clinical Relevance:**

The cryptococcosis has different presentations, it´s a disease whose incidence has been increasing since the advent of the HIV / AIDS pandemy, however the commitment of the encephalic parenchyma and in particular the cerebellum is considered rare. In this way we are facing the first case of cryptococcal cerebellitis in our midst.

## Resumen

**Introducción:**

La Criptococosis es una infección micótica oportunista cuya etiología es el complejo *Cryptococcus neofromans/C. gattii*, el cual principalmente afecta pacientes inmunocomprometidos. La afección meníngea es una de las formas más frecuentes pero el compromiso cerebeloso es raro.

**Descripción del Caso:**

Paciente masculino de 65 años, procedente de un área rural con exposición crónica a aves de corral, sin antecedentes patológicos, con cuadro clínico inicial consistente en cefalea crónica, fiebre, convulsiones y alteración del estado mental.

**Hallazgos clínicos y métodos diagnósticos:**

Al principio sin signos de hipertensión intracraneana ni meníngeos y examen neurológico normal, con posterior desarrollo de ataxia, disdiadococinesia y dismetría. Se diagnosticó Cerebelitis Criptocococica con ayuda de repetidos estudios de LCR y resonancia magnética nuclear.

**Tratamiento:**

Se inició terapia antifúngica con Anfotericina B y Fluconazol, con respuesta tórpida y el paciente fallece.

**Relevancia clínica:**

La Cerebelitis Criptocococica es una presentación clínica infrecuente que requiere sospecha clínica y recursos diagnósticos para definir el tratamiento de forma temprana. La inmunosupresión no es requisito para padecer esta infección.

## Introduction

Cryptococcosis is a fungal infection, whose etiology is the complex *Cryptococcus neofromans / C. Gattii*. It is considered a sentinel disease, and Human immunodeficiency Virus (HIV) is the most common coinfection ^1^. This mycosis is acquired by inhalation of basidiospores found in bird excreta and tree detritus. The neurotropism characteristic of this fungus is attributed to urease and laccase enzymes, by which it synthesizes prostaglandin E and melanin, which allow it to survive inside the macrophages and through these cross the blood-brain barrier, mechanism known as the Trojan horse ^2^.

Global incidence varies between 0.04-12.0% annually and Sub-Saharan Africa is considered the region with the highest number of estimated cases in the world ^3^. Cryptococcal meningitis is the most common clinical form, it has an incidence of more than 1 million cases and 600 thousand deaths annually. A significant proportion of patients with cerebral cryptococcosis are immunocompetent. In these cases, *C. gattii* infection is more frequent, which is considered to be highly adaptive to changes in ecological conditions ^4^.

The following is a case of cryptococcus cerebellitis, in a patient without history of importance that presented a diagnostic challenge due to its nonspecific clinical manifestations.

## Case presentation

A 65-year-old male, a farmer, from a subtropical area south of the department of Cauca, located in the Colombian Massif, with an average environmental temperature of 19° C. He works in continuous contact with poultry and has no previous history of importance. He consulted for intense holocranial headache of four months of evolution that yielded partially with NSAIDs. Forty-eight hours before admission he was exacerbated with nausea and generalized clonic seizures of the subintrant type. He was treated with a dose of intravenous loading of Valproic Acid, continued with 500 mg every 8 hours, which controlled the seizures during the time of hospitalization. 

**Figure 1:**
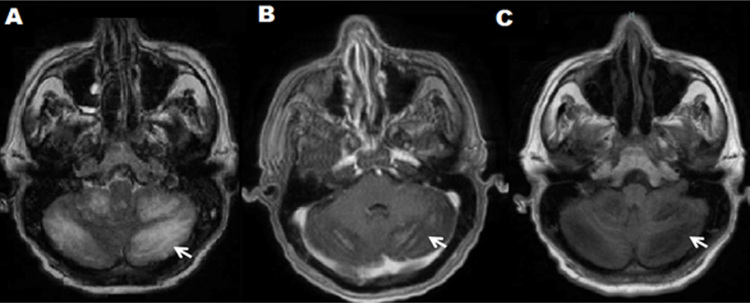
A-B-C: Contrasted NMR. A: Axial cut T2 Axial Flair; Bilateral predominantly right diffuse hyperintense cerebellar lesion. B: Diffuse hypointense cerebellar lesion with marked contrast enhancement, in Axial T1 cut plus contrast. C : Diffuse hypointense cerebellar lesion without evidence of nodule or abscess, in axial Axial T1 Flair.

A neurological examination revealed an obscure, confused patient with normal fundoscopy, no signs of endocranial hypertension, nor meningeal, normal force in all four limbs. A determination of Reactive Protein C and Globular Sedimentation Velocity was performed, a hemogram with leukocytosis and neutrophilia. The ELISA test for HIV was negative. With a diagnostic impression of epileptic syndrome, a computerized axial tomography (CT) scan of the brain was requested and showed no signs of endocranial hypertension, expansive lesions, or other relevant lesions. A lumbar puncture was performed demonstrating hypoglycemia and hyperproteinorraquia (Table 1), so it was decided to study as meningitis. On the third day of hospitalization, the patient persisted with altered state of consciousness, and presented dysmetria, dysarthria, dysdiadochokinesia, a positive Stewart-Holmes sign and a right hand drive without nystagmus, nor intentional tremor. For persisting without clinical improvement, another lumbar puncture was performed, which found that he found a high opening pressure, pleocytosis and hypoglycemia. The Chinese ink staining allowed to identify encapsulated blastoconidides compatible with *Cryptococcus* spp that were confirmed in cultures in blood agar, chocolate and Sabouraud, where *C. neoformans var. Grubii NIV* in the Laboratory of the National Institute of Health of Colombia. Antifungal therapy with Amphotericin B at 50 mg daily and Fluconazole at 400 mg every 12 hours was initiated, with improved mental status, decreased febrile episodes, headache, ataxia and dysmetria. On the ninth day of hospitalization, the results of the MRI were reported: in the T1 segment diffuse hypointense cerebellar lesion with contrast enhancement without evidence of nodule or abscess; In the T2 Flair sequence, a predominantly right bilateral diffuse hyperintense cerebellar lesion corresponding to inflammatory changes was evidenced. (Fig. 1A, B, C). The definitive diagnosis of Cryptococcal Cerebellum was made. The same antifungal therapy was continued for one week until epileptic status was reported for a period of approximately 12 min and died.

**Table 1:** Behavior of the characteristics of the CSF on the time of hospitalization.

	Study day 1	Study day 4	Reference values
Appearance	Slightly turbid	Slightly turbid	Clear
Cell records (cells /mm3)	Negative	13	0-5 mononuclear
Glucose ( mg/dl)	11	5	40-80
Proteins ( mg/dl)	112.8	73.0	15-45
Lactate ( mmol/l)	10.2	9.1	1-2
Bacyloscopy	Negative	Negativo	Negative
Chinese ink	Negative	Encapsulated blastoconidia compatible with *Cryptococcus spp.*	Negative
Staining papanicolau		Positive	Negative
Intracranean pressure ( cm/H_2_0)		25	10-15

## Discussion

Due to the relationship between this mycosis, HIV / AIDS infection and other forms of immunosuppression, cryptococcosis in immunocompetent patients can be considered exceptional ^1^. Another associated entity is the selective CD4 + idiopathic lymphocytopenia syndrome, described since the 80's, which predisposes the patient to present from lung infection by atypical mycobacteria to meningeal cryptococcosis, the latter caused mostly by C. neoformans ^5^ ^,^ ^6^ .

In the presented case, the integrity of the patient's immune system could be affected by age (immunosenescence) ^7^. During aging there are alterations in the functional and quantitative level of the components of the immune system that lead to a higher incidence of autoimmune diseases and infections mainly by encapsulated microorganisms, pathologies that indicate the presence of a poorly efficient immune system. Changes such as inversion of the lymphocyte ratio CD4 +: CD8 +; which is generally 2: 1 in healthy adults, due to the relative increase in CD8 + lymphocyte counts; In addition to decreased numbers of neutrophils and increased numbers of Natural Killer cells (NK); as well as functional changes such as reduced response of mononuclear cells, especially T-lymphocytes, and changes in the structure of Toll-like receptors, all favoring the onset of pathologies characteristic of immunocompromised patients ^7^ ^,^ ^8^. Due to the acute course of this patient, the measurement of parameters such as CD4 and CD8 counts was unrealizable.

In this case the infection occurred in a rare anatomical site, the cerebellum. This type of compromise is rare and poorly reported, despite this being the most frequent mycosis of the central nervous system. Compared with other reports this case has many similarities. Most were in immunocompetent patients, chronic and progressive headache associated with nausea and vomiting was the main symptom during the onset of the disease. One case had cerebellar symptoms such as ataxia and dysdiadochokinesia ^9^; in another case the alteration of the state of consciousness was one of the first symptoms ^10^, and in another no meningeal signs were reported ^11^.

The clinical presentation of this case differs, due to the absence of cryptococomas, which are solid lesions, mainly located in basal ganglia or cerebral white matter, corresponding to abscesses or granulomas, which can be detected by NMR; ^12^, neoplasms of the central nervous system ^13^, brain abscesses ^14^ and even tuberculomas ^15^ with diagnostic histopathological examination after surgical management.

Meningeal cryptococcosis is manifested clinically by headache, nausea, vomiting, fever and only one-third of those affected show signs of spinal root irritation, this is more frequent in immunocompetent patients. Neurological examination may include: alteration of the mental sphere, decreased strength in extremities, convulsions, involvement of cranial nerves (II, VI, VII, VIII). In the fundus of the eye you may see optic atrophy and often papilledema. The most frequent sequels are visual alterations such as blindness, which is the most common, besides deafness, paresis of extremities and language disorder. Meningeal signs such as occurred in our case are not very eloquent, this causes the diagnosis to be avoided and a clinical synergy is necessary with the contribution of neuroimaging, laboratory findings and epidemiological considerations. In this sense, authors such as Brizendine et al. ^16^, Yi-Chien et al. ^17^, Lizarazo et al. ^18^ have compared the clinical presentation, CSF and radiological findings among immunocompetent and immunocompetent patients, agreeing that HIV positive patients tend to present more frequently CSF parameters such as glycemia, Proteinuria, and pleocytosis within normal ranges compared to HIV-negative patients. The clinical and radiological parameters are not consistent.

New molecular biology techniques such as the enzyme immunoenzyme (EIA) and Polymerase Chain Reaction (PCR) with sensitivity and specificity of 100% for both tests, which allow to classify 8 new serotypes of *Cryptococcus Spp*, and also the subsequent typing of the therapeutic responses of each of these, which may contribute to improve the prognosis of this infection ^19^ ^,^ ^20^.

Infection by *Cryptococcus spp* of the CNS has higher mortality than other presentations of this disease and being even higher by *C. gatti*. The factors most associated with poor prognosis are: altered mental status, CSF opening pressure greater than 25 cm of water, cryptococcal antigen in CSF greater than 1: 512, culture positive for cryptococcus in organs other than the CNS, and absence of meningeal enhancement ^6^. The patient in this case presented three: altered mental state, persistently high opening pressure and absence of meningeal enhancement.

## Conclusion

In this case, an immunocompetent patient presents with signs of cerebellar compromise. The lack of specificity of the clinical manifestations and the low detection rate of Cryptococcus in the CSF pose difficulties for its diagnosis, so it is necessary to perform multiple CSF studies for its identification, in addition to a radiological study, especially by magnetic resonance imaging, to make a definitive diagnosis.
