# Supplementary material for: Cryptococcal cerebellitis in no-VIH patient
Source: Colomb Med (Cali). 2017 Jun 30;48(2):94–7. (PMC5625563)
Supplement: Translation

# Translation: Cerebelitis criptococócica en paciente VIH negativo

## Introducción

La criptococosis es una infección micótica cuya etiología es el complejo *Cryptococcus neofromans/C. gattii*. Se considera una enfermedad centinela, y la coinfección más frecuente es con el Virus de la Inmunodeficiencia Humana (VIH) ^1^. Esta micosis se adquiere mediante inhalación de basidioesporas que se encuentran en excretas de aves y detritus de árboles. El neurotropismo característico de este hongo se atribuye a las enzimas ureasa y lacasa, mediante las cuales sintetiza prostaglandina E y melanina, que le permiten sobrevivir dentro de los macrófagos y a través de estos atravesar la barrera hematoencefálica, mecanismo conocido como el caballo de Troya ^2^.

La incidencia global varía entre 0.04-12.0% anual y se considera el África Subsahariana como la región con mayor número de casos estimados a nivel mundial ^3^. El compromiso meníngeo es la forma clínica más común, tiene una incidencia mayor a 1 millón de casos y ocasiona 600 mil muertes al año. Una importante proporción de pacientes con criptococosis cerebral son inmunocompetentes, en estos casos es más frecuente la infección por C. gattii, el cual se considera una especie altamente adaptativa a variaciones de las condiciones ecológicas ^4^. 

A continuación se presenta un caso de cerebelitis criptocococica, en un paciente sin antecedentes de importancia que supuso un gran reto diagnóstico por sus manifestaciones clínicas inespecíficas.

## Presentación de caso

Paciente masculino de 65 años, agricultor, procedente de una zona subtropical al sur del departamento del Cauca, ubicada en el Macizo Colombiano, con una temperatura ambiental media de 19° C. Trabaja en contacto continuo con aves de corral y no tiene otros antecedentes de importancia. Consultó por cefalea holocraneana intensa de cuatro meses de evolución, que cedía parcialmente con AINE. Cuarenta y ocho horas antes del ingreso se exacerbó con náuseas y crisis convulsivas tónicas clónicas generalizadas de tipo subintrantes. Fue tratado con una dosis de carga endovenosa de Ácido Valproico, se continuó con 500 mg cada 8 horas, qué controló las convulsiones durante el tiempo de la hospitalización. Al examen neurológico se encontró un paciente obnubilado, confuso, con fundoscopia normal, sin signos de hipertensión endocraneana, ni meníngeos, fuerza normal en sus cuatro extremidades. Se realizó determinación de Proteína C Reactiva y Velocidad de Sedimentación Globular que se encontraron elevadas, un hemograma con leucocitosis y neutrofília. El examen de ELISA para VIH fue negativo. Con una impresión diagnóstica de síndrome epiléptico, se solicitó una tomografía axial computarizada (TAC) de cerebro que no mostró signos de hipertensión endocraneana, lesiones expansivas, ni otras lesiones relevantes. Se realizó una punción lumbar que demostró hipoglucorraquia e hiperproteinorraquia (Tabla 1), por lo que se decidió estudiar como meningitis. Al tercer día de hospitalización, el paciente persistió con alteración del estado de conciencia, además presentó dismetría, disartria, disdiadococinesia, signo de Stewart-Holmes positivo y latero pulsión derecha sin nistagmo, ni temblor intencional. Por persistir sin una mejoría clínica, se hace realizó otra punción lumbar que encontró una presión de apertura elevada, pleocitosis e hipoglucorraquia. La tinción de tinta china permitió identificar blastoconididas encapsuladas compatibles con *Cryptococcus spp* que fueron confirmadas en cultivos en agar sangre, chocolate y Sabouraud, donde se aisló *C. neoformans var. grubii VNI* en el Laboratorio del Instituto Nacional de Salud de Colombia. Se inició terapia antifúngica con Anfotericina B a 50 mg día y Fluconazol a 400 mg cada 12 horas, con mejoría del estado mental, disminución de los episodios febriles, cefalea, ataxia y dismetría. Al noveno día de hospitalización se reportaron los resultados de la RMN: en la secuencia T1 lesión cerebelosa hipointensa difusa con realce al contraste, sin evidencia de nódulo ni absceso; en la secuencia T2 Flair se evidenció una lesión cereberelosa hiperintensa difusa bilateral de predominio derecho que corresponden a cambios inflamatorios. (Fig. 1 A, B, C). Se realizó el diagnóstico definitivo de Cerebelitis Criptocococica. Se continuó con la misma terapia antifúngica durante una semana hasta que se presentó status epiléptico por un periodo de aproximadamente 12 min y falleció.

**Tabla 1:** Comportamiento de las características del LCR sobre el tiempo de hospitalización.

	Estudio Día 1	Estudio Día 4	Valores de Referencia
Apariencia	Ligeramente turbio	Ligeramente turbio	Claro
Recuento celular (cells /mm3)	Negativo	13	0-5 mononucleares
Glucosa ( mg/dL)	11	5	40-80
Proteínas ( mg/dL)	112.8	73.0	15-45
Lactato ( mmol/L)	10.2	9.1	1-2
Baciloscopia	Negativo	Negativo	Negativo
Tinta china	Negativo	Blastoconidias encapsuladas compatibles con *Cryptococcus spp*	Negativo
Tinción Papanicolau		Positivo	Negativo
Presión intracraneal (cm/H_2_O)		25	10-15

**Figura 1:** A- B-D: RMN Contrastada. A: RMN en corte Axial T2 Flair; lesión cereberelosa hiperintensa difusa bilateral de predominio derecho. B: Lesión cerebelosa hipointensa difusa con marcado realce con el contraste, en corte Axial T1 más contraste. C: Lesión cerebelosa hipointensa difusa sin evidencia de nódulo ni absceso, en corte Axial T1 Flair.

## Discusión

Debido a la relación existente entre esta micosis, la infección por VIH/SIDA y otras formas de inmunosupresión, la criptococosis en pacientes inmunocompetentes se puede considerar excepcional ^1^. Otra entidad asociada es el síndrome de Linfocitopenia idiopática CD4+ selectiva, descrita desde los años 80's, que predispone a quien la padece a presentar desde infección pulmonar por micobacterias atípicas hasta criptococosis meníngea, ésta última causada en su mayoría por *C. neoformans* ^5^^,^^6^.

En el caso presentado la integridad del sistema inmune del paciente pudo afectarse por la edad (inmunosenescencia) ^7^. Durante el envejecimiento se dan alteraciones a nivel funcional y cuantitativo de los componentes del sistema inmune que llevan a una mayor incidencia de enfermedades autoinmunes e infecciones principalmente por microorganismos encapsulados, patologías que indican la presencia de un sistema inmune poco eficiente. Se dan cambios como la inversión de la relación de linfocitos CD4+:CD8+; que generalmente es 2:1 en adultos sanos, debido al aumento relativo del conteo de linfocitos CD8+; además de disminución del número de neutrófilos y aumento en número de las células Natural Killer (NK); de igual manera, cambios funcionales como una menor respuesta de las células mononucleares especialmente de los linfocitos T, y cambios en la estructura de los receptores Toll Like, todos estos favorecen a la aparición de patologías propias de pacientes con inmunocompromiso ^7^^,^^8^. Debido al curso agudo de este paciente resultó irrealizable la medición de parámetros como el conteo de CD4 y CD8. 

En este caso la infección ocurrió en un sitio anatómico poco frecuente, el cerebelo. Este tipo compromiso es raro y escasamente reportado, a pesar de ser esta entidad la micosis más frecuente del sistema nervioso central. Comparado con otros reportes este caso tiene muchas similitudes. La mayoría se presentaron en pacientes inmunocompetentes, la cefalea crónica y progresiva asociada a nausea y vómito, fue el principal síntoma durante el debut de la enfermedad. Un caso presentó síntomas cerebelosos como ataxia y disdiadococinesia ^9^; en otro caso la alteración del estado de conciencia fue uno de los primeros síntomas ^10^ y en otro tampoco se reportaron signos meníngeos ^11^. 

La presentación clínica de este caso difiere, por la ausencia de criptococomas, que son lesiones sólidas, localizados principalmente en ganglios basales o sustancia blanca cerebral, que corresponden a abscesos o granulomas, que pueden detectarse por RMN; en algunos casos se plantearon diagnósticos diferenciales como malformaciones vasculares ^12^, neoplasias del sistema nervioso central ^13^, abscesos cerebrales ^14^ e incluso tuberculomas ^15^ con diagnostico al examen histopatológico posterior al manejo quirúrgico.

La criptococosis meníngea se manifiesta clínicamente por cefalea, náuseas, vómito, fiebre y solo un tercio de los afectados presenta signos de irritación de raíces raquídeas, esto es más frecuente en pacientes inmunocompetentes. La exploración neurológica puede encontrar: alteración de la esfera mental, disminución de la fuerza en extremidades, convulsiones, compromiso de pares craneales (II, VI, VII, VIII). En el fondo de ojo se puede ver atrofia óptica y con frecuencia papiledema. Las secuelas más frecuentes son alteraciones visuales como ceguera, la cual es la más común, además de sordera, paresia de extremidades y trastorno del lenguaje. Los signos meníngeos tal como ocurrió en nuestro caso son poco elocuentes, esto hace que el diagnóstico se soslaye y es necesaria una sinergia clínica con el aporte de neuroimágenes, hallazgos de laboratorio y las consideraciones epidemiológicas. En este sentido, autores como Brizendine *et al*. ^16^, Yi-Chien *et al*. ^17^, Lizarazo *et al*. ^18^, han comparado la presentación clínica, hallazgos del LCR y radiológicos entre pacientes inmunocomprometidos e inmunocompetentes, coincidiendo en que los pacientes VIH positivos tienden a presentar con mayor frecuencia parámetros del LCR como la glucorraquia, proteínorraquia y pleocitosis dentro de rangos normales en comparación con pacientes VIH negativos. Los parámetros clínicos y radiológicos no son consistentes. 

Nuevas técnicas de biología molecular como el inmunoenzayo enzimático (EIA por sus siglas en inglés) y Reacción en Cadena de Polimerasa (PCR por sus siglas en inglés) con sensibilidad y especificidad del 100% para ambos test, que permiten clasificar 8 nuevos serotipos de *Cryptococcus spp*, y además la posterior tipificación de las respuestas terapéuticas de cada una de éstas, lo cual podrá contribuir a mejorar el pronóstico de ésta infección ^19^^,^ ^20^.

La infección del SNC por *Cryptococcus spp* tiene mayor mortalidad que otras presentaciones de esta enfermedad y siendo aún más alta por *C. gatti.* Los factores más asociados a peor pronóstico son: alteración del estado mental, presión de apertura LCR mayor a 25 cm de agua, antígeno criptocócico en LCR mayor a 1:512, cultivo positivo para criptococo en órganos diferentes al SNC y ausencia de realce meníngeo ^6^. El paciente de este caso presentó tres: alteración del estado mental, presión de apertura persistentemente alta y ausencia de realce meníngeo. 

## Conclusión 

En este caso, un paciente inmunocompetente se presenta con signos de compromiso cerebelar. La falta de especificidad de las manifestaciones clínicas y la baja tasa de detección del Criptococo en el LCR plantean dificultades para su diagnóstico, por lo que es necesario realizar múltiples estudios del LCR para su identificación, además de un estudio radiológico, especialmente por resonancia magnética, para hacer un diagnóstico definitivo.
